# Stroma-Targeting Therapy in Pancreatic Cancer: One Coin With Two Sides?

**DOI:** 10.3389/fonc.2020.576399

**Published:** 2020-10-15

**Authors:** Bolun Jiang, Li Zhou, Jun Lu, Yizhi Wang, Chengxi Liu, Lei You, Junchao Guo

**Affiliations:** Department of General Surgery, Peking Union Medical College Hospital, Chinese Academy of Medical Sciences and Peking Union Medical College, Beijing, China

**Keywords:** pancreatic ductal adenocarcinoma, stroma, chemotherapy, tumor microenvironment, nanotherapy

## Abstract

Pancreatic ductal adenocarcinoma (PDAC) is a malignancy with one of the worst prognoses worldwide and has an overall 5-year survival rate of only 9%. Although chemotherapy is the recommended treatment for patients with advanced PDAC, its efficacy is not satisfactory. The dense dysplastic stroma of PDAC is a major obstacle to the delivery of chemotherapy drugs and plays an important role in the progression of PDAC. Therefore, stroma-targeting therapy is considered a potential treatment strategy to improve the efficacy of chemotherapy and patient survival. While several preclinical studies have shown encouraging results, the anti-tumor potential of the PDAC stroma has also been revealed, and the extreme depletion might promote tumor progression and undermine patient survival. Therefore, achieving a balance between stromal abundance and depletion might be the further of stroma-targeting therapy. This review summarized the current progress of stroma-targeting therapy in PDAC and discussed the double-edged sword of its therapeutic effects.

## Introduction

Pancreatic ductal adenocarcinoma (PDAC) ranks seventh as the cause of cancer-associated mortality worldwide and has an overall 5-year survival rate of only 9% (1, 2). In the United States, PDAC is projected to become the second deadliest cancer by 2030 (3). With the acceleration of industrialization and population aging, the incidence of PDAC has also increased in China (4). In the past few decades, the treatment of PDAC has not made substantial progress. Surgery remains the only treatment that might achieve a cure. However, about 80% of PDAC patients are unable to access surgery at the time of diagnosis (5). Because of the early recurrence and metastasis of PDAC, even the patients who have already undergone radical surgery present a 5-year survival rate of only 25% (5). Therefore, PDAC has emerged as a major public health problem that needs to be addressed urgently.

For the majority of patients with advanced PDAC, chemotherapy is the generally recommended treatment. The standardized chemotherapy regimens include the use of the cytosine nucleoside analog gemcitabine or a more potent but highly toxic four-drugs regimen, FOLFIRINOX (i.e., 5-fluorouracil, leucovorin, oxaliplatin, and irinotecan) (6). In addition, chemotherapy is also applied as a neoadjuvant treatment for some PDAC patients who are suitable for surgery to lower the risk of recurrence (7). However, the efficacy of these regimens is not satisfactory. One of the main features of PDAC is its dense abundant stroma, which increases the interstitial fluid pressure (IFP). The compression of intratumoral vasculature impedes the efficiency of chemotherapeutic agent delivery and forms a hypoxic microenvironment, which promotes tumor progression (8, 9). Therefore, reversing the negative effects of PDAC stroma might improve the efficacy of chemotherapy and patient survival (8).

## PDAC Stroma and Its Role in Disease Progression

In PDAC, tumor cells account for less than 20% of the total tumor volume, while the stroma components occupy more than 70% (10). The dense desmoplasia stroma of PDAC consists of several cellular components (e.g., fibroblasts, stellate cells, immune cells, and pericytes), acellular components (e.g., fibrin, collagen, hyaluronic acid, fibronectin, growth factors, and cytokines), and biophysical components (e.g., low pH, hypoxia, and high tumor IFP) (11). These components interact mutually to promote the progression of PDAC (12).

During the progression of PDAC, pro-inflammatory cytokines secreted by neoplastic cells stimulate fibroblasts and pancreatic stellate cells (PSCs), which produce extracellular matrix (ECM) and increase fibrotic stromal deposition (13, 14). The solid stress generated by the dense stroma and lymphatic obstruction causes intratumoral IFP to increase, which leads to vascular compression, tissue perfusion reduction, and a hypoxic microenvironment (15, 16). In fact, approximately 80% of intratumoral blood vessels are dysfunctional, poorly fenestrated, and covered with a thick layer of pericytes, which hinder the effective accumulation of chemotherapeutic agents. In addition, the PDAC stroma potentially alters the pharmacokinetics and pharmacodynamics of chemotherapeutic drugs (8). For example, the high expression level of cytidine deaminase in the stroma shortened the circulating half-life of gemcitabine (17). Furthermore, the fibrotic microenvironment of PDAC results in an inhibitory effect on the innate and adaptive immune systems, reducing cytotoxic T cells and increasing M2 macrophages, N2 neutrophils, and T-regulatory cells (Tregs) at tumor sites. Growth factors and cytokines secreted by PSCs also promote the formation of a tumor immunosuppressive microenvironment (18). In summary, the dense proliferative stroma of PDAC promotes tumor progression and metastasis *via* various routes (11). Therefore, overcoming the tumor-promoting effects of PDAC stromal barriers has become an imperative issue.

## Stroma-Targeting Therapy in PDAC

In order to overcome the physical and biological barriers to effective PDAC treatments, several strategies based on targeting the stroma have been designed to improve the efficacy of chemotherapeutic agents and reverse the stroma’s impact on tumor progression (**Table 1**).

**Table 1:** Clinical trials assessing stroma-targeting therapy in PDAC.

Target	Agents	Patient population	Trial phase	mPFS (months)	mOS (months)	Status	NIH number
HA	PEGPH20 + GEM + nab-paclitaxel vs GEM + nab-paclitaxel	Metastatic PDAC	Phase II	6.0 vs 5.3	9.6 vs 9.2	Completed	NCT01839487
	PEGPH20 + GEM + nab-paclitaxel vs GEM + nab-paclitaxel	Metastatic PDAC (HA-High)	Phase II	9.2 vs 5.2	11.5 vs 8.5	Completed	NCT01839487
	PEGPH20 + GEM + nab-paclitaxel vs GEM + nab-paclitaxel	Metastatic PDAC (HA-High)	Phase III	7.1 vs 7.1	11.2 vs 11.5	Terminated	NCT02715804
	PEGPH20 + FOLFIRINOX vs FOLFIRINOX	Metastatic PDAC	Phase Ib/II	4.3 vs 6.2	7.7 vs 14.4	Terminated	NCT01959139
Hedgehog	IPI-926	Solid tumors (including PDAC)	Phase I	–	–	Completed	NCT00761696
	IPI-926 + GEM vs GEM	Metastatic PC	Phase Ib/II	The former < the latter	The former < the latter	Completed	NCT01130142
	IPI-926+ FOLFIRINOX	Locally advanced or metastatic PDAC	Phase Ib	8.4	–	Completed	NCT01383538
	Vismodegib + GEM vs GEM	Metastatic PC	Phase Ib/II	4.0 vs 2.5	6.9 vs 6.1	Completed	NCT01064622
	Vismodegib +GEM	Metastatic PC	Phase II	2.8	5.3	Completed	NCT01195415
	Vismodegib +GEM + nab-paclitaxel	Metastatic PDAC	Phase II	5.42	9.79	Completed	NCT01088815
	Sonidegib + GEM	Locally advanced or metastatic PDAC	Phase Ib	4.9	–	Completed	NCT01487785
CTGF	Pamrevlumab + GEM + nab-paclitaxel vs GEM + nab-paclitaxel	Locally advanced PDAC	Phase I/II	–	–	Completed	NCT02210559
	Pamrevlumab + GEM + nab-paclitaxel vs GEM + nab-paclitaxel	Locally advanced PDAC	Phase III	–	–	Recruiting	NCT03941093
	Pamrevlumab + GEM + erlotinib	Locally advanced or metastatic PDAC	Phase I	4.3	9.4	Completed	NCT01181245
R-A system	Losartan + FOLFIRINOX + chemoradiotherapy	Locally advanced PDAC	Phase II	17.5	31.4	Completed	NCT01821729
	Losartan + nivolumab + FOLFIRINOX + SBRT	Localized PDAC (Borderline/potentially resectable or locally advanced)	Phase II	–	–	Recruiting	NCT03563248
–	UM + GEM	Locally advanced or metastatic PDAC	Phase I	–	17.6 vs 8.9	Completed	NCT01674556

CTGF, connective tissue growth factor; DDR1, discoidin domain receptor 1; FOLFIRINOX, 5-fluorouracil, leucovorin, oxaliplatin, and irinotecan; GEM, gemcitabine; HA, hyaluronic acid; NIH, National Institutes of Health; mOS, median overall survival; PC, pancreatic cancer; PDAC, pancreatic ductal adenocarcinoma; mPFS, median progression-free survival; R-A system, renin-angiotensin system; SBRT, stereotactic body radiation therapy; UM, ultrasound microbubbles.

### Depletion of the Stroma in PDAC

**Figure 1:**
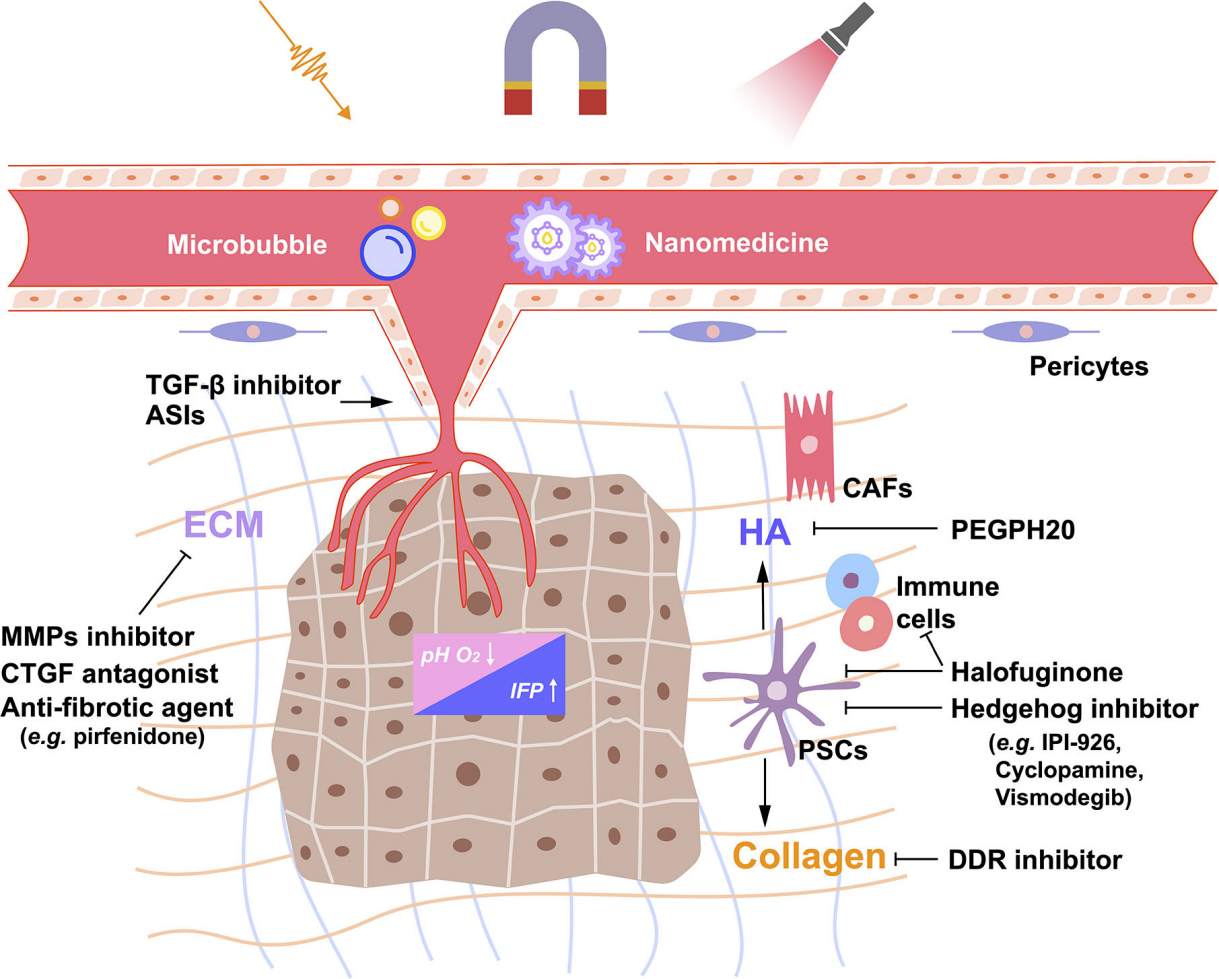
Schematic of the extracellular matrix network and stroma-targeting strategies in pancreatic ductal adenocarcinoma (PDAC). The dense desmoplasia stroma of PDAC consists of several cellular, acellular and biophysical components, which interact mutually to promote the growth and metastasis of PDAC. Inactivating pancreatic stellate cells (PSCs) or a certain population of cancer-associated fibroblasts (CAFs) by Hedgehog inhibitors and halofuginone or altering the products these CAFs produce, such as hyaluronic acid (HA) and collagen, by PEGPH20 or discoidin domain receptor (DDR) inhibitor might be potential strategies to reverse the dense dysplastic stroma of PDAC. In addition, remodeling the tumor vasculature by transforming growth factor-β (TGF-β) inhibitors or angiotensin system inhibitors (ASIs) decreases the interstitial fluid pressure (IFP), reverses acidosis, and the hypoxic environment, and enhances the efficacy of drug delivery. Furthermore, nano-drug delivery systems have been designed to respond to environmental or external stimuli which triggers drug release. T-mark represents inhibition; arrow, activation. ECM, extracellular matrix; MMPs, matrix metalloproteinase; CTGF, connective tissue growth factor.

One of the main components of PDAC stroma is hyaluronic acid (HA) or hyaluronan (**Figure 1**). HA is a complex glycosaminoglycan secreted abundantly by neoplastic cells (19–21), and it is a core polymer in the assembly of multiple hydrophilic matrix proteoglycans (22). HA binds to cell surface receptors to maintain tumor cell survival and activate downstream signaling pathways related to tumor proliferation, migration, and invasion (23–25). In addition, its ability to absorb and retain water increases IFP (24, 26). Therefore, HA is considered a potential therapeutic target in PDAC. Pegylated hyaluronidase (PEGPH20) is a pegylated nanoscale complex of recombinant human hyaluronidase (27, 28). Previous studies showed that PEGPH20 induced the degradation of HA, remodeled tumor vasculature, and improved chemotherapeutic drug efficacy (22, 27, 29). A phase II clinical study HALO-109-202 (NCT01839487), involving 279 patients with metastatic PDAC, showed that PEGPH20 combined with Abraxane (an albumin-bound paclitaxel nanocomplex) and gemcitabine nearly doubled the progression-free survival and improved the overall survival in patients with high level HA (30). However, a subsequent phase III clinical study HALO-109-301 (NCT02715804) showed that PEGPH20 combined with Abraxane and gemcitabine did not significantly extend the overall survival of PDAC patients. Therefore, Halozyme announced the termination of further research and development of PEGPH20 in November 2019.

Another major component of the tumor ECM is ​​collagen (**Figure 1**). PDAC stroma is rich in fibrillar collagens, which support tumor cell survival and promote tumor progression through discoidin domain receptor 1 and 2 (DDR1 and 2). High expression of DDR1 was identified as an independent risk factor for poor prognosis in patients with PDAC (31). In fact, the selective small molecule inhibitor of DDR1 reduced collagen deposition and improved the response to chemotherapy in PDAC mouse models (32). Preclinical studies are currently investigating the effects of DDR1 inhibition on the immune microenvironment of PDAC. Recently, the potent antifibrotic ability of halofuginone (a natural quinazolinone alkaloid febrifugine analog) was observed in a genetically engineered mouse model of PDAC (33). Halofuginone directly inhibited the activation of PSCs, thereby reducing the deposition of several ECM components, including collagen and HA. The decrease of the intratumoral IFP resulted in the improved delivery of chemotherapeutic agents, which was visually proven by exploiting the autofluorescence of doxorubicin. In addition, halofuginone helped increase immune cell infiltration and shift them closer to cytokeratin positive tumor cells, suggesting that stroma-targeting therapy might improve the efficacy of immunotherapy to some extent in PDAC.

The Hedgehog (Hh) signaling pathway is generally hyperactive in PDAC (**Figure 1**) (34). It is known that the Hh signaling pathway activates PSCs through paracrine effects and regulates stroma deposition (35). There are several strategies intended to treat PDAC by inhibiting the Hh signaling pathway in order to eliminate the tumor stroma (36). Cyclopamine is a natural steroidal alkaloid that inhibits the Hh signaling pathway *via* binding to Smoothened (SMO) (37). It reduced fibronectin content and enhanced tumor vascularization in a PDAC xenograft mouse model. When combined with paclitaxel-loaded nanoparticles, cyclopamine increased tumor growth inhibition by 63.3% (38). In addition, a preclinical study showed that IPI-926, a small molecule that inhibited the Hh pathway, increased blood vessel density and gemcitabine drug concentration in PDAC (39). In a phase I clinical trial, IPI-926 was well tolerated by PDAC patients (40). However, a phase II clinical trial of IPI-926 failed to show significant therapeutic benefits in PDAC patients (41, 42). Similarly, Vismodegib, another Hh pathway inhibitor, combined with gemcitabine did not significantly improve the median overall survival and progression-free survival in patients with metastatic PDAC compared with gemcitabine alone (43). In fact, while several treatments targeting the Hh signaling pathway showed uplifting results in preclinical models (44), few of them improved patient survival, and their use was often accompanied by drug-related toxicity (45). In addition to the above-mentioned drugs, other stroma-targeting drugs include metalloproteinase inhibitors (46–48), connective tissue growth factor (CTGF) antagonists (e.g., pamrevlumab) (49, 50), and anti-fibrotic agents (e.g., pirfenidone) (**Figure 1**) (51, 52).

### Remodeling Tumor Vasculature

The dense fibrotic stroma surrounding the blood vessels and the proliferating cancer-associated fibroblasts (CAFs) damage intratumoral vasculature, resulting in a hypoxic microenvironment, which promotes PDAC invasion, metastasis, and the acquisition of chemoresistance phenotypes (**Figure 1**) (53–55). Therefore, remodeling PDAC blood vessels not only improves drug delivery but also overcomes the hypoxic microenvironment.

The transforming growth factor-β (TGF-β) signaling pathway is involved in the adhesion of pericytes to tumor vascular endothelial cells (56). TGF-β receptor kinase inhibitors or monoclonal antibodies enhanced vascular access and promoted the perfusion of chemotherapeutic agents to PDAC tumor sites (56–58). In addition, the repurposing of antihypertensive drugs targeting the renin-angiotensin pathway can reprogram CAFs towards normalizing ECM by inhibiting TGF-β (59, 60). In tumors, Angiotensin II (Ang II) activates TGF-β and promotes CAFs to express CTGF by binding to Ang II type 1 receptor (AT1R) (61). Moreover, Ang II stimulates proliferation of PSCs by activating protein kinase C and EGF-ERK signaling pathways (61, 62). Several angiotensin system inhibitors (ASIs) have been used to target the PDAC stroma (59, 63). A phase II clinical study evaluated the efficacy of neoadjuvant chemoradiotherapy with FOLFIRINOX combined with losartan in patients with locally advanced PDAC. The therapy was associated with a high proportion (69%) of patients achieving R0 resection (64). Interestingly, the lack of Angiotensin II type 2 receptor (AT2R) in pancreatic fibroblasts also leads to tumor cell proliferation (65). Therefore, the role of the Ang II signaling pathway in the PDAC stroma needs to be further investigated before its use in the clinic.

In addition to the pharmacological interference, focused ultrasound combined with microbubbles was investigated to determine if it improved vascular patency. Microbubbles are restrained within the vascular compartment because of their diameter. Focused ultrasound causes the microbubbles to disintegrate, which releases acoustic forces that are capable of inducing thrombolysis and increasing drug penetration (66). Moreover, appropriate ultrasound energy induces the destruction of the microbubbles and generates acoustic pressure that temporarily increases the permeability of blood vessels and cell membranes (66). The efficacy of ultrasound microbubble delivery of gemcitabine was evaluated in a PDAC murine model. The study showed that the ultrasound microbubbles treatment group presented significant tumor growth inhibition (67). In addition, the use of nab-paclitaxel combined with ultrasound microbubbles significantly inhibited the activity of PDAC tumor cells *in vitro* and reduced the tumor volume in a subcutaneous PDAC mouse model (68). Furthermore, a clinical trial evaluated the efficacy of focused ultrasound combined with microbubble delivery of gemcitabine in PDAC patients. The combination-treated patients tolerated more gemcitabine chemotherapeutic cycles, and half of them showed maximum tumor diameter decrease. In addition, the median survival was prolonged by 8.7 months (69).

### Nano-Drug Delivery Systems in PDAC

The dense stroma of PDAC limits the application of chemotherapeutic drugs to efficiently target neoplastic cells through the enhanced permeability and retention (EPR) effect (15, 70). Nanomedicine, the result of the formulation of drugs into nano-size delivery carriers, such as liposomes and polymer nanoparticles, delivers chemotherapeutic agents through mechanisms that are different from those used to deliver conventional non-encapsulated molecular drugs (71). By optimizing the particle size, surface charge, and specific ligand modification of nanocarriers, nanomedicine improves the ability of nanocarriers to penetrate the stromal barrier in PDAC tumor sites (72). A study showed that both 30-nm and 100-nm nanoparticles penetrated into the hyperpermeable colon cancer model, but only the 30-nm nanoparticle penetrated the stroma-rich PDAC model and achieved anti-tumor efficacy in the murine model (73). Different charges on ECM components make the nano-drug delivery heterogeneous in spatial distribution, compromising the drug delivery efficiency (74). Therefore, active targeted drug delivery with modified specific ligands has been designed to address the issues (75, 76). Several studies showed that nanoparticle albumin–bound paclitaxel (Nab-paclitaxel) improved the delivery efficiency of paclitaxel. In addition, nab-paclitaxel combined with gemcitabine improved the overall survival and disease-free survival in patients with advanced PDAC (77), and this combination therapy strategy has been approved by the Food and Drug Administration (FDA). Several nab-paclitaxel-based combination therapy strategies are currently in the clinical evaluation stage (78, 79). A recent meta-analysis showed that nanoparticles improved the efficacy of various anti-tumor drugs while reducing their toxicity in patients with PDAC (80).

To further improve the efficiency of nano-delivery drugs, smart nanoparticles have been designed to respond to environmental or external stimuli, which triggers drug release after passive or active tumor accumulation (81). The PDAC tumor microenvironment exhibits some unique physiological characteristics, such as the low pH, cancer cells lysosomes (81), and a high level of reducing substances in tumor cells (e.g., glutathione) (82). Therefore, a nano-drug delivery system responsive to the stimuli from the internal environment of the tumor has been designed. In addition, researchers have also designed nano-drug delivery systems triggered by the external stimuli. Thermal, magnetic, and mechanical waves (including ultrasound) *via* image guidance have been applied to trigger the nano-drug delivery system to release the loaded drug. Furthermore, a study combined the internal and external stimuli and designed a new nano-drug carrier to respond to a three-dimensional trigger (ultrasonic, acid-sensitivity and reduced glutathione) (**Figure 1**) (83).

## Stroma-Targeting Therapy as a Double-Edged Sword

The tumor suppressive properties of the PDAC stroma have been revealed gradually. Several studies, using genetic or pharmacological approaches to eliminate the PDAC stroma in preclinical models, showed that stromal depletion promoted tumor cell proliferation, invasion, and metastasis and reduced survival (84, 85). PDAC tumor cells in the primary lesion showed loss of differentiation, epithelial-to-mesenchymal transition (EMT), and enhanced cancer stem cell-like phenotypes after stromal ablative treatment. In addition, severe weight loss, acidosis, and cachexia were observed in PDAC mouse models (84–87). These findings suggest that stroma depletion might activate dormant neoplastic cells and induce their metastatic potential, thereby promoting PDAC progression.

Prominently, different subtypes of CAFs that exhibit distinct characteristics and activity levels have been identified in breast cancer (88) and PDAC (89). In PDAC, CAFs have been classified into several subpopulations on the basis of the expression patterns of various fibroblast markers (89, 90). One subgroup is characterized by the α-SMA expression and generally considered as myofibroblastic CAFs, while another is classified as inflammatory CAFs for the secretion of multiple inflammatory chemokines (e.g., IL-6). Studies have assessed the effects of these CAFs subgroups on tumor microenvironment and immune escape in PDAC. For example, the targeted inhibition of IL-6, which is produced by inflammatory CAFs, improved the efficacy of anti-PD-L1 checkpoint inhibitors in PDAC. However, the genetic deletion of Hedgehog resulted in the reduction of stroma deposition and myofibroblastic CAFs, inducing more aggressive and de-differentiated tumor phenotypes (85). Similarly, another study determined that genetic deletion of myofibroblastic CAFs induced more aggressive PDAC tumor phenotypes, manifested by EMT, cancer stemness, chemotherapy resistance, and tumor immune evasion (84). These findings suggest that inflammatory CAFs might be the potential therapeutic target, while myofibroblastic CAFs support tumor suppression in PDAC. Recently, Biffi et al. identified that TGFβ and interleukin 1 (IL1) as tumor-secreted ligands promoted CAFs heterogeneity, illuminating strategies to selectively target CAFs that support tumor growth (91).

Pericyte is an important cellular component related to the tumor vasculature in PDAC (92). One of the main biomarkers associated with vascular pericytes is platelet-derived growth factor receptor-β (PDGFR-β) (93). Its ligand, PDGF, was shown to induce pericyte-fibroblast transition (PFT), which makes pericyte an origin of CAFs, thereby promoting tumor invasion and metastasis. Pharmacological inhibition and genetic deletion of PDGFR-β reversed PDGF induced PFT (92). However, PDGFR-β inhibitors (e.g., imatinib and sunitinib) caused pericytes to detach from vascular endothelial cells, destroying vasculature integrity and promoting tumor metastasis (93). Therefore, non-discriminatory depletion of CAFs might remove partial tumor-suppressing CAFs subgroups. Although stroma-targeting therapy enhances the delivery of chemotherapeutic agents, it might also promote tumor chemoresistance and metastasis. In fact, the unsatisfactory results in clinical applications and the revelation that the PDAC stroma harbors tumor suppressive activity indicates that future research should focus on the tumor ECM biology. The various components in the PDAC stroma might have different effects on tumor biological behavior. Therefore, the pathophysiological analysis of distinct PDAC stromal cell might enlighten future stroma-targeting strategies (90).

## Discussion and Perspective

The dense desmoplasia stroma of PDAC, which hinders the efficient delivery of chemotherapeutic agents, plays a critical role in tumor progression and metastasis. On the one hand, various stroma-targeting treatment strategies have been applied to improve drug efficacy and inhibit tumor progression. However, the clinical efficacy of these treatments has not been satisfactory in PDAC patients. On the other hand, several studies showed that extreme PDAC stromal depletion caused a deterioration in tumor phenotypes, supporting the perspective that the stromal response is a host response to inhibit tumor growth. Therefore, a stroma-targeting treatment might not be an optimal neoadjuvant therapy for patients with locally advanced PDAC.

It is worth noting that lack of taking the heterogeneity of PDAC stromal composition into account might be one reason why unsatisfactory results were observed in the clinical studies. Stroma-targeting therapy would be beneficial in sites with high density of the targeted stromal composition, while be a disadvantage in sites with low density. Therefore, it is crucial to access the quantitative degree and type of tumor stroma density (TSD), which might be a significant section of the clinical study design (94). The phase II study, HALO-109-202, was an example of stratification analysis based on the level of HA (30). In addition, the activation status of stroma could be identified, according to consensus clustering of expression levels of certain biomarkers (95). For instance, prior work found that A Disintegrin And Metalloproteases 12 (ADAM12) was a blood-borne proxy for stromal activation, the levels of which had prognostic significance and correlate with treatment benefit (96). In fact, the stroma is a reservoir of various potential biomarkers, which might be used to classify PDAC patient subgroups and facilitate the clinical application of stroma-targeting therapy.

In addition, the heterogeneity of stromal microenvironment between the primary tumor and metastases sites might result in distinct responses to stroma-targeting therapy (94). Previous studies showed that PDAC metastatic tumors had limited driver-mutation heterogeneity compared to the primary tumors (97), and TSD was various across metastatic sites and primary tumors (94). These observations suggested that the heterogeneity of stroma might be a function of host organ-site physiology and not inferior to cancer genomic or epigenomic variations between the primary and metastatic tumors. Furthermore, the effects of individual and ethnic-specific genetic variations on the stroma microenvironment of PDAC might need further investigation. Genomic analyses of single PDAC stroma cell might help identify inter- and intra-individual heterogeneity and optimize individualized stroma-targeting therapy in order to maximize patient treatment benefits (98–100).

Pharmacological agents that alter the tumor microenvironment might help evade the negative effect of stromal depletion. For example, the inhibition of PDAC tumor-associated macrophages by clodronate liposomes prevented the establishment of a pre-metastatic microenvironment in organs of metastasis, markedly reducing metastasis formation (101). Therefore, clodronate liposomes might be a candidate for combination strategies with stroma-targeting therapy. Furthermore, although the proliferative stroma of PDAC usually exerts an immunosuppressive effect, stromal depletion might exacerbate the immunosuppression effect in some cases (84). Therefore, the combination of immune checkpoint inhibitors and stroma-targeting therapy might reduce the negative effects of stromal ablation and further enhance the efficacy of chemotherapy in PDAC patients.

In summary, stroma-targeting therapy in PDAC has emerged as a double-edged sword. On the one hand, it enhances the delivery of chemotherapeutic agents to tumor sites. On the other hand, it eliminates the physical and biochemical barriers that potentially inhibit tumor progression. Therefore, it is imperative to understand the complex role of the PDAC stroma in order to improve the current stroma-targeting therapies through appropriately adjusting the balance between stroma deposition and ablation.

## Author Contributions

BJ and JG managed the study design. BJ, JL, and YW conducted the literature search. CL performed the data extraction. BJ drafted the manuscript. LZ, LY, and JG reviewed the manuscript and made critical revisions for important intellectual content. JG provided funding support. All authors contributed to the article and approved the submitted version.

## Funding

The present study was supported by the National Natural Science Foundation of China (Grant No. 81972324) and the China Academy of Medical Sciences Innovation Fund for Medical Sciences (Grant No. 2016-I2M-3-019).

## Conflict of Interest

The authors declare that the research was conducted in the absence of any commercial or financial relationships that could be construed as a potential conflict of interest.
